# Significance and Biological Importance of Pyrimidine in the Microbial World

**DOI:** 10.1155/2014/202784

**Published:** 2014-03-23

**Authors:** Vinita Sharma, Nitin Chitranshi, Ajay Kumar Agarwal

**Affiliations:** ^1^School of Pharmacy, Lloyd Institute of Management & Technology, Plot. No. 11, Knowledge Park II, Greater Noida, Uttar Pradesh 201306, India; ^2^Bioinformatics Centre, Biotech Park, Sector G, Jankipuram, Lucknow, Uttar Pradesh 226021, India; ^3^Gautam Buddh Technical University, IET Campus, Sitapur Road, Lucknow, Uttar Pradesh 226021, India; ^4^Department of Pharmaceutical Sciences, University Institute of Pharmaceutical Sciences, Kurukshetra University, Kurukshetra, Haryana 136119, India

## Abstract

Microbes are unique creatures that adapt to varying lifestyles and environment resistance in extreme or adverse conditions. The genetic architecture of microbe may bear a significant signature not only in the sequences position, but also in the lifestyle to which it is adapted. It becomes a challenge for the society to find new chemical entities which can treat microbial infections. The present review aims to focus on account of important chemical moiety, that is, pyrimidine and its various derivatives as antimicrobial agents. In the current studies we represent more than 200 pyrimidines as antimicrobial agents with different mono-, di-, tri-, and tetrasubstituted classes along with *in vitro* antimicrobial activities of pyrimidines derivatives which can facilitate the development of more potent and effective antimicrobial agents.

## 1. Introduction

Resistance to antimicrobial agents has become an increasingly important and pressing global problem. Of the 2 million people who acquire bacterial infection in US hospitals each year, 70% of cases now involve strains that are resistant to at least one drug [1]. In communities and hospitals around the world, the number of patients with antibiotic-resistant infections continues to climb [2]. A major cause for concern in the UK is methicillin-resistant* Staphylococcus aureus* (MRSA), which was at low levels a decade ago but now accounts for ca. 50% of all* S. aureus* isolates [3]. Substantial investment and research in the field of anti-infectives are now desperately needed if a public health crisis is to be averted. The causes of antimicrobial resistance are multifactorial. In case of an antibiotic, it has been well documented that resistance is mainly caused by continued overreliance on and imprudent use of these antibacterial agents [4] and increasing evidence is being obtained suggesting that the same may be true for the emergence of biocide resistance [5, 6]. Of particular concern is the possible cross-resistance of antibiotics and biocide due to common resistance mechanism [7, 8]. Metal resistance is being observed as the result of polluted environments [9, 10]. The consequence of continued exposure to antibacterial environment is an enrichment of bacteria that are intrinsically resistant to antimicrobials or have acquired resistance mechanism to these substances [11, 12].

Structural modification of antimicrobial drugs to which resistance has developed has been proven to be an effective means of extending the lifespan of antifungal agents such as the azoles [13], antiviral agents such as the nonnucleoside reverse transcriptase inhibitors [14], and various antibacterial agents including *β*-lactams and quinolones [15]. It is not surprising then that, in response to antimicrobial resistance, major pharmaceutical companies have tended to concentrate their efforts on improving antimicrobial agents in established classes [16]. However, with the portfolio of chemotherapeutics currently available, it has been acknowledged that researchers are getting close to the end game in terms of parent structure alterations. A call has therefore been made for the development of new classes of drugs that work on different target sites to those in current use [17, 18].

**Figure 1:**
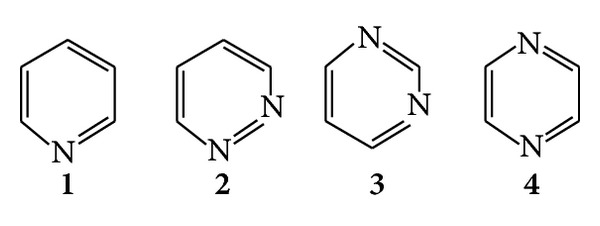
Pyridine and different isomeric forms of diazine family.

Heterocyclic compounds are abundant in nature and are of great significance to life because their structural subunits exist in many natural products such as vitamins, hormones, and antibiotics [19, 20]; hence, they have attracted considerable attention in the design of biologically active molecules [21, 22] and advanced organic chemistry [23, 24]. Also in the family of heterocyclic compounds nitrogen containing heterocycles are an important class of compounds in the medicinal chemistry and also contributed to the society from biological and industrial point which helps to understand life processes [25]. A totally unsaturated membered six-ring containing nitrogen is known as azine [26] or pyridine (**1**); with two nitrogen atoms it is known as diazine [27], and with a nitrogen at 1,2-position, it is known as pyridine (**2**), at 1,3-position as pyrimidine (**3**), and at 1,4-position as pyrazine (**4**) (Figure 1). However, the current review intends to focus on the significance of pyrimidine class of antimicrobial agents along with clinical and* in vitro* applications of pyrimidine derivatives to facilitate the development of more potent as well as effective antimicrobial agents.

## 2. Pyrimidine: General Introduction

**Figure 2:**
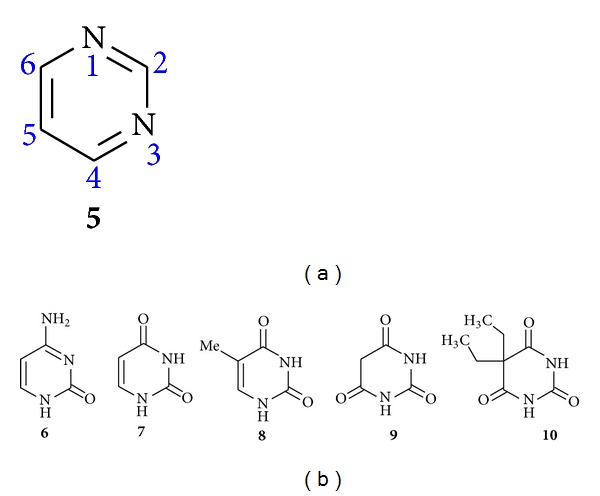
(a) Pyrimidine. (b) Pyrimidine containing natural and synthetic products.

Pyrimidines (**5**) are the heterocyclic aromatic compounds similar to benzene and pyridine containing two nitrogen atoms at positions 1 and 3 of the six membered rings. Heterocycles containing pyrimidine moiety are of great interest because they constitute an important class of natural and nonnatural products, many of which exhibit useful biological activities and clinical applications [28, 29]. Substituted purines and pyrimidines occur very widely in living organisms and were some of the first compounds studied by the organic chemists [30] (Figure 2(a)).

Pyrimidines are biologically very important heterocycles and represent by far the most ubiquitous members of the diazine family with uracil (**6**) and thymine (**7**) being constituents of ribonucleic acid (RNA) and deoxyribonucleic acid (DNA) and with cytosine (**8**) both being present in Figure 2(b).

In addition to this, pyrimidines skeleton is also present in many natural products such as vitamin B_1_ (thiamine) and many synthetic compounds, such as barbituric acid (**9**) and Veranal (**10**) which are used as hypnotics [31] (Figure 2(b)).

## 3. Medicinal Properties of Pyrimidines

The presence of pyrimidine base in thymine, cytosine, and uracil, which are the essential building blocks of nucleic acids DNA and RNA, is one possible reason for their widespread therapeutic applications. The pyrimidines represent one of the most active classes of compounds possessing wide spectrum of biological activities like significant* in vitro *activity against unrelated DNA and RNA, viruses including polioherpes viruses, diuretic, antitumour, anti-HIV, and cardiovascular [32]. The literature survey indicated that a wide range of pharmacological activities are exhibited by the compounds encompassing pyrimidines nucleus. In addition to this, various analogs of pyrimidines have been found to posses antibacterial [33–39], antifungal [40–43], antileishmanial [44], anti-inflammatory [45, 46], analgesic [47], antihypertensive [48, 49], antipyretic [50], antiviral [51–53], antidiabetic [54], antiallerggic [55], anticonvulsant [56], antioxidant [57, 58], antihistaminic [59], herbicidal [60], and anticancer activities [61–64] and many of pyrimidines derivatives are reported to possess potential central nervous system (CNS) depressant properties [65, 66] and also act as calcium channel blockers [67].

## 4. Clinical and Pharmacological Applications of Pyrimidine in the Microbial World: Marketed Drugs

During the last two decades several pyrimidine derivatives have been developed which are found to have wideclinical and pharmacological applications [68].

### 4.1. Antibacterial Agents

Drugs which are included in this category are antifolates possessing antagonistic activity against folic acid and sulfa drugs which are sulphur containing pyrimidine derivative drugs.

#### 4.1.1. Antifolates

2-Amino-4-hydroxypyrimidines are found to be antagonists of folic acid [69]; hence, a large number of 2,4-diaminopyrimidines have been synthesized as antifolates and it was eventually proved that these pyrimidines are inhibitors dihydrofolate reductase (DHFR) [70, 71]. Notable amongst the 2,4-diaminopyrimidine drugs are the following.

**Figure 3:**
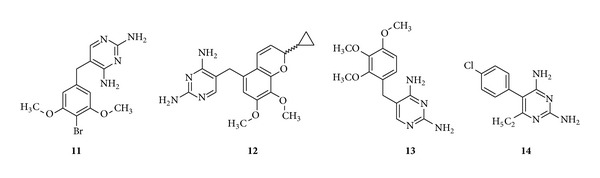
Antibacterial drugs (antifolates) containing trisubstituted and tetrasubstituted pyrimidines.

*Trisubstituted Pyrimidine Containing Drugs*. Brodiprim (**11**) is found to be an effective antibacterial compound [72]. Iclaprim (**12**) which is a new selective dihydrofolate inhibitor was synthesized based on rational drug design and this drug is found to be active against methicillin-, TMP-, and vancomycin-resistant strains [73, 74]. Trimethoprim (**13**) is an antibacterial drug which selectively inhibits bacterial DHFR [75] (Figure 3).

*Tetrasubstituted Pyrimidine Containing Drug*. Pyrimethamine (**14**) is a selective inhibitor of the DHFR of malarial plasmodia [75] (Figure 3).

#### 4.1.2. Sulfa Drugs

Pyrimidine containing sulfa drugs are classified on the basis of substitution and the classification with the respective example of drug is as follows.

**Figure 4:**
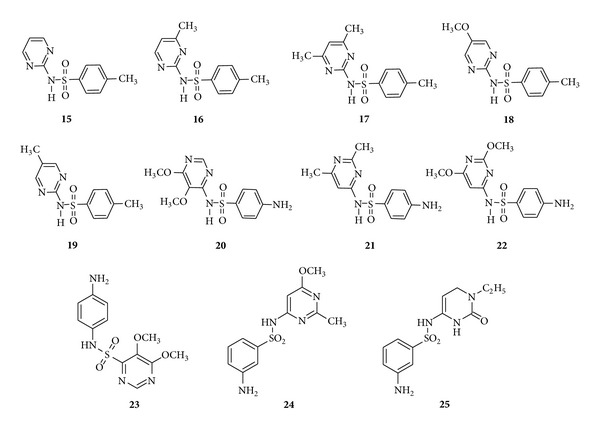
Sulfa drugs having monosubstituted (**15**), disubstituted (**16**–**19**), and trisubstituted (**20**–**25**) pyrimidine.

*Monosubstituted and disubstituted sulfa drugs *include sulfadiazine (**15**), sulfamerazine (**16**), sulfadimidine (sulfamethazine) (**17**), sulfameythoxydiazine (**18**), and methyldiazine (**19**) (Figure 4). Sulfadiazine, sulfamerazine, and sulfadimidine are superior to other sulfonamides and are used in some acute bacterial infections, cerebrospinal meningitis, and patients allergic to penicillins [68]. Sulfameythoxydiazine (**18**) possesses a good half life and methyldiazine (**19**) possesses a longest half life of about 65 h [76].

*Trisubstituted sulfa drugs *include sulfadoxine (**20**), sulfisomidine (**21**), sulfadimethoxine (**22**), sulfamethoxine (**23**), sulfamethomidine (**24**), and sulfacytine (**25**) (Figure 4).

Sulfadoxine which has a half life of 7–9 days is used for malarial prophylaxis [77] whereas sulfisomidine with a half life of 7 hr is used as a combination sulfa therapy in human serum hemolytic complement system [78]. Sulfadimethoxine was introduced in 1959 with a half life of approximately 40 hr and the related 4-sulfamidopyrimidine and sulfamethoxine, having two methoxy groups in 5 and 6 positions, have by far the longest half life of about of 65 hr. Further, a new broad-spectrum sulfonamide, sulfamethomidine, is relatively nontoxic and patients do need extra fluid to intake and sulfacytinehas been reported to be 3–10 times more potent than sulfaisoxazole and sulfisomidine [76].

Sulfa drugs are known to possess good antibacterial activity in combination with trimethoprim [2,4-diamino-5-(3,4,5-trimethoxy benzyl pyrimidine)], resulting in synergistic effect [79]. Sulfonamide-trimethoprim combinations are used extensively for opportunistic infections in patients with Acquired Immunodeficiency Syndrome (AIDS) [80].

### 4.2. Antibiotics

Antibiotics containing pyrimidines moiety are further classified on the basis of substitution on the ring.

**Figure 5:**
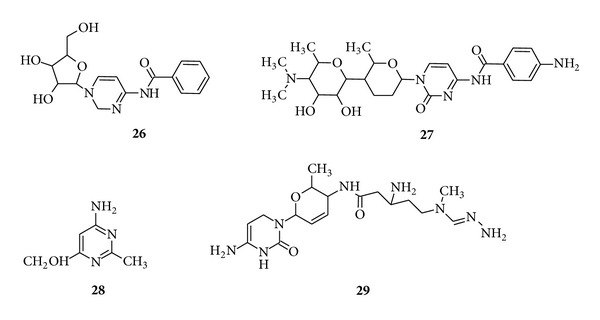
Antibiotics representing disubstituted (**26**) pyrimidine moiety and trisubstituted (**27**–**29**) pyrimidine moiety.

*Disubstituted Pyrimidine Containing Antibiotic Drug*. Amicetin (**26**) is cytosine derivative which exhibits activity against acid fast and Gram-positive bacteria as well as some other organisms [81] (Figure 5). 

*Trisubstituted Pyrimidine Containing Antibiotic Drug*. Plicacetin (**26**) is another cytosine derivative which exhibits activity against bacteria [81]. The simplest pyrimidine antibiotic of all is bacimethrin (**28**) (5-hydroxy-2-methoxy-pyrimidin-4-amine) which is active against several staphylococcal infections [81]. Gourgetin (**29**) is a cytosine derivative which is active against mycobacteria as well as several Gram-positive and Gram-negative bacteria (Figure 5). Aminoglycoside antibiotics phleomycin, bleomycin and related families are wide-spectrum antibiotics containing the ring pyrimidine ring [82]. Bleomycin (see Figure S1 in Supplementary Material available online at http://dx.doi.org/10.1155/2014/202784) is already in clinical use against certain tumours like Hodgkin's lymphoma and disseminated testicular cancer [83].

### 4.3. Antivirals and Anti-HIV (AIDS) Agents

**Figure 6:**
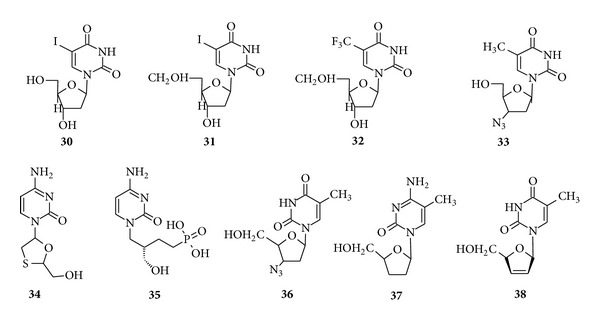
Antiviral drugs having pyrimidine moiety.

Pyrimidines derivatives also possess good antiviral properties; for example, 5-iododeoxyuridine (**30**) and IDU (5-iodo-2′-deoxyuridine) (**31**) have been extensively utilized for viral infections. 5-Trifluoromethyl-2′-deoxyuridine (**32**) has been found useful against infections resistant to IDU therapy [84]. 1-(3-Azido-2,3-dideoxypentofuranosyl)-5-methyl-2,4(1*H*,3*H*)-pyrimidinedione (**33**) is a potent inhibitor of the* in vivo* replication and cytopathic effects of HIV and has been approved for use against AIDS and severe AIDS-Related Complex (ARC) [85] (Figure 6).

Lamivudine (**34**) is an effective anti-AIDS when used in combination with zidovudine; cidofovir (**35**) is used for the treatment of* cytomegalovirus* (CMV) [86]. Zidovudine (**36**) which is an analogue of thymidine in which the azido group is substituted at the 3-position of the dideoxyribose moiety is active against RNA tumour viruses (retroviruses) that are the causative agents of AIDS and T-cell leukaemia. Zalcitabine (**37**) is another useful drug and is given in combination with Zidovudine [87]. Stavudine (**38**), a pyrimidine nucleoside, is found to show significant activity against HIV virus when given in combination with zidovudine [88].

### 4.4. Antifungal Drugs

**Figure 7:**
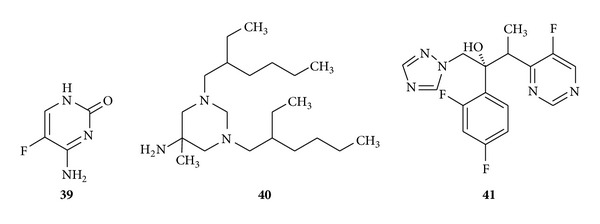
Pyrimidine containing antifungal drugs.

Pyrimidines also exhibit antifungal properties (see Figure 7). Flucytosine (**39**) is a fluorinated pyrimidine and is an orally active antifungal agent which is used for the treatment of serious systemic infections caused by susceptible strains of* Candida* and* Cryptococcus* [89, 90] and hexetidine (**40**) is mainly used for the treatment of aphthous ulceration [91]. Recently, voriconazole (**41**) is a disubstituted drug undergoing phase *Ι* *Ι* *Ι* comparative clinical trials to establish its full potential as a broad-spectrum antifungal agent [92, 93].

## 5. *In Vitro* Antimicrobial Activity Profile of Pyrimidines: Literature Review

In the past few years great advances have been made in the synthesis of novel pyrimidines derivatives with a potent antimicrobial activity. The following review will examine the* in vitro* antimicrobial activity of pyrimidines, a class of heterocyclic compounds possessing a diverse range of pharmacological properties. The* in vitro *antimicrobial activity is further divided into antibacterial, antifungal, antimycobacterial, antituberculosis, antileishmanial, antiamoebic, antiparasitic, and antiviral activities on the basis of its activity against the particular microorganisms and these activities are briefly discussed in turn, with particular emphasis on pyrimidine moiety along with their SAR studies (structure activity relationships).

### 5.1. Antibacterial Activity and Antifungal Activity

This is the first category to be described here which consists of pyrimidine derivatives possessing activity against the various strains of bacteria and fungi. These various pyrimidine derivatives were further divided on the basis of substitution on the pyrimidine ring and this classification is as follows.

#### 5.1.1. Monosubstituted and Disubstituted Pyrimidine Derivatives

**Figure 8:**
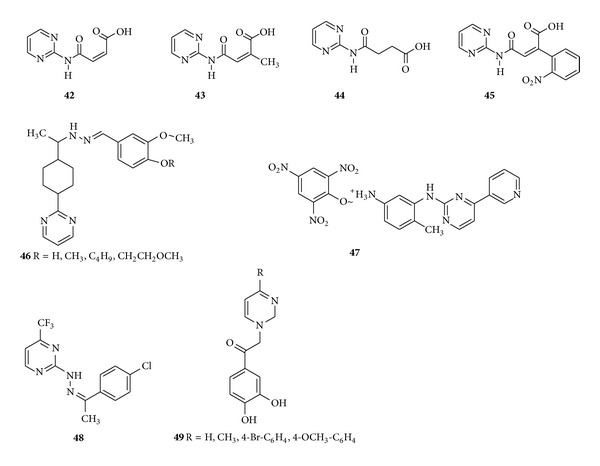
Monosubstituted pyrimidine derivatives with antibacterial and antifungal activity.

Kolotova et al. synthesized dicarboxylic acid 2-pyrimidylamides derivatives and evaluated the* in vitro* antibacterial activity of the synthesized compounds with respect to the standard strains of* E. coli* and* S. aureus*. The compounds (**42**–**45**) exhibited an* in vitro* antibacterial activity against all the tested strains with MIC value equal to 500–1000 *μ*g/mL [94]. Hydrazone derivatives of vanillin are found to possess potent antibacterial activity. Based on higher bioactivity of hydrazones, new hydrazone derivatives (**46**) were synthesized from piperidine-4-carboxylic acid methyl ester and checked for antibacterial activities by paper diffusion method by measuring the zone of inhibition in millimeters against* Pseudomonas aeruginosa *and* Staphylococcus aureus* bacterial strains and compared with standard drugs ciprofloxacin and cefaclor. The zone of inhibition of tested compounds shows the vanillin coupled hydrazone derivatives encompass potent bioactivities against bacterial strains [95] (Figure 8).

Pyrimidine salt such as 4-methyl-3-(4-pyridin-3-yl-pyrimidin-2-ylamino)-phenyl ammonium-2,5-dichloro-4-hydroxy-3,6-dioxo-cyclohexa-1,4-dienolate (**47**) chloranilic acid was synthesized and screened for* in vitro* antibacterial activity. Results revealed that the above compound exhibited good activity with the zone of inhibition in the range of 16 mm against pathogenic bacterial strains and good antifungal activity with the 56.4% inhibition against* F. oxysporum* when compared with the standard drug [96] (Figure 8).

In search of new* T. cruzi* inhibitor, Zanatta et al. in 2008 synthesized a series of novel 2-(*N*′-benzylidinehydrazino)-4-trifluoromethyl pyrimidines derivatives and evaluated them to inhibit the growth of* T. cruzi*. The compound (**48**) was found to be the most active compound and was able to produce most significant inhibitory effect (80%) and presented an IC_50_ value of 85 *μ*M [97]. Balan et al. synthesized some new diazinium salts with dihydroxyacetophenone and screened them for their antibacterial activity against* S. aureus*,* S. saprophyticus*,* Sarcina lutea*,* B. subtilis*,* B. cereus*,* E. coli*, and* P. aeruginosa* and antifungal activity against* C. albicans* and found that the compound (**49**) showed excellent antibacterial and antifungal activity which is much more than that of chloramphenicol and almost similar to that of nystatin [98] (Figure 8).

#### 5.1.2. Trisubstituted Pyrimidine Derivatives

**Figure 9:**
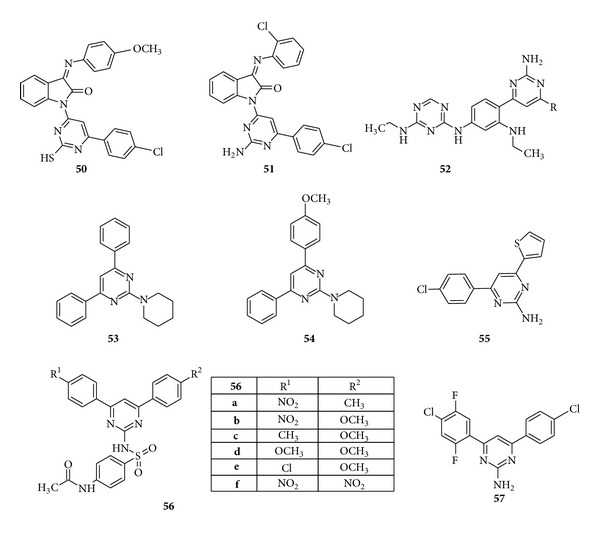
Trisubstituted pyrimidine derivatives of antibacterial and antifungal activity.

A novel series of mercaptopyrimidine and aminopyrimidine derivatives of indoline-2-one were synthesized by Mondal et al. and screened for their* in vitro* antibacterial activity against Gram-positive bacteria* Staphylococcus aureus*,* Bacillus subtilis *and Gram-negative bacteria* Salmonella typhi*,* Shigella dysenteriae*,* Pseudomonas mirabilis*, and* Escherichia coli*. It was found that compounds (**50**) and (**51**) with a methoxy (OCH_3_) substitution on phenyl ring at* para* position and hydroxyl (–OH) group at ortho position were found potent and (–Cl) substitution showed a very good zone of inhibition at concentration of 100 *μ*g/mL against both Gram-positive and Gram-negative bacteria, comparable to standard drug Ampicillin [99]. Further derivatives of aminopyrimidines were synthesized by Solankee et al. and evaluated for their* in vitro* antibacterial activity against various bacterial strains. Compound (**50**) was found to show small amount of activity against* S. aureus* [100]. Sukhwal and Verma synthesized the novel series of 2-piperidin-4,6-diaryl substituted pyrimidines by carrying out the reaction between the S-benzylisothiouronium chloride (SBT) with variously substituted chalcones and evaluated the synthesized compounds for their* in vitro *antibacterial activity. Results revealed that the compounds (**53**) and (**54**) showed the maximum to moderate activity against* Klebsiella pneumonia*,* Pseudomonas vulgaris*,* E. coli*, and* Pseudomonas aeruginosa *[101]. A novel series of 2-amino-4-(4′-chlorophenyl)-6-(substituted)-pyrimidines were synthesized by Desai et al. and they evaluated the compounds for* in vitro *antibacterial activity. Results revealed that the compound (**55**) showed the highest antibacterial activity against* E. coli*,* Salmonella *(Gram −ve) and* Bacillus pumilus*,* micrococcus* (Gram +ve) [102] (Figure 9).

Wasfy and Aly reported some new derivatives of diphenylysulfapyrimidine acetates synthesized by the condensation of sulfaguanidine acetate with chalcones bearing an electron withdrawing group. Results revealed that all the compounds were weakly or moderately active and the compound (**56**) having combination of substituents like Cl, OCH_3_, and NO_2_ possessed fairly marked activity whereas compound (**56f**) bearing 2-nitro group (electron withdrawing group) had activity comparable to commercial antibacterial agent sulfadiazine [103]. With the view of physiological importance and pharmaceutical properties of 2-amino pyrimidines, Devi et al. synthesized some derivatives 2-amino-4-(2′,4′-dichloro,5′ fluro)-6-phenyl pyrimidine and evaluated them for* in vitro *antibacterial activity. Results showed that the compound (**55**) having chloro group showed moderate activity on all strains of bacteria [104] (Figure 9).

**Figure 10:**
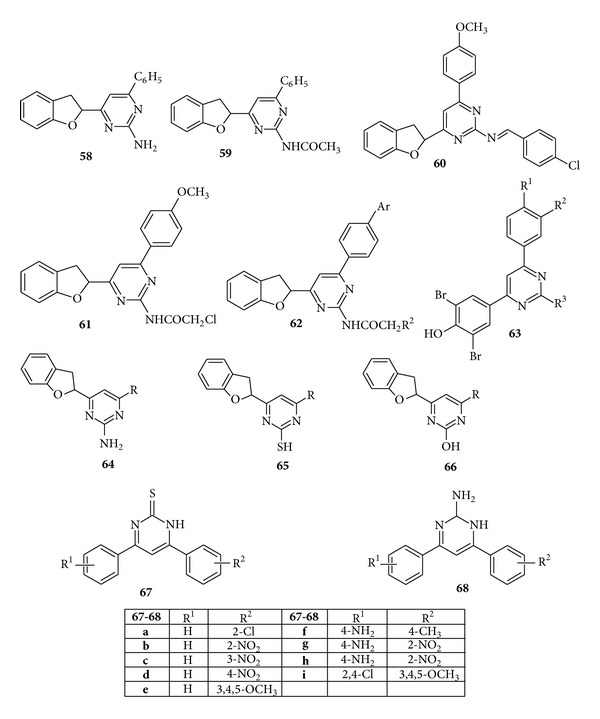
Trisubstituted pyrimidine derivatives of antibacterial and antifungal activity.

Some more derivatives of pyrimidines having benzofuryl ring were reported by Yadav et al. who evaluated the* in vitro* antimicrobial activity of the compounds. It was found that the compounds (**58**–**62**) showed good activity against* B. subtilis*,* E. coli*,* A. niger*, and* C.albicans *[105]. Kothari et al. synthesized some new 2-(1-piperidinyl/1-pyrrolidinyl)-4-substituted phenyl-6 (3′,5′-dibromo-4′-hydroxy phenyl)-pyrimidine derivatives and evaluated the synthesized compounds against Gram-positive organisms* Streptococcus viridians *and* S. aureus *and Gram-negative organisms* E. coli *and* K. pneumonia*. The compound (**63**) was found to display moderate activity [106] (Figure 10).

Some substituted pyrimidines derivatives containing benzofuran have been synthesized by Babu et al. and screened for the* in vitro* antimicrobial activity. Results revealed that the compounds (**64**–**66**) if suitably substituted were found to show antibacterial and antifungal activity, that is, against* P. aeruginosa*,* S. aureus*, and* C. albicans* [107]. Pasha et al. prepared 4,6-disubstituted pyrimidine-thiones and 2-amino-4,6-disubstituted pyrimidines by interaction of various chalcones and evaluated them for* in vitro *antibacterial and antitubercular activities and it was found that compounds (**67**) and (**68**) showed moderate activity against* B. subtilis*,* S. aureus*,* E. coli*,and* Pseudomonas aeruginosa* [108] (Figure 10).

**Figure 11:**
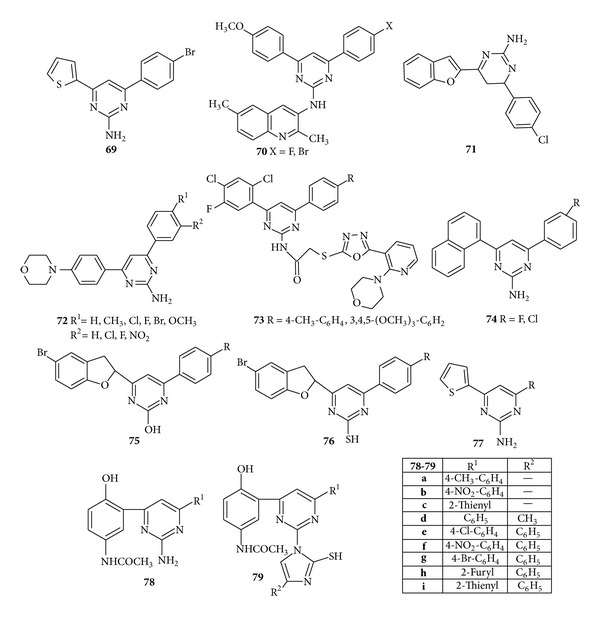
Trisubstituted pyrimidine derivatives of antibacterial and antifungal activity.

Chandrasekharan and Nagarajan synthesized some novel 2-amino-6-aryl-4-(2-thienyl) pyrimidines and evaluated the compounds for* in vitro *antibacterial activity. Results revealed that the halogen substituted 4-fluoro, that is, compound (**69**), was found to be more active than 4-chloro, 4-bromo and the rest of other compounds [109]. Novel pyrimidines quinoline clubbed molecules were synthesized by Patel and Mehta who studied their* in vitro *antibacterial activity of all the synthesized compounds against the* S. aureus*,* Streptococcus pyogenes*,* E. coli*, and* P. aeruginosa* and antifungal activity against* C. albicans*,* A. niger*. Results showed that the presence of methoxy and halogen groups (Cl, Br, and F) in the phenyl ring of the compound (**70**) increases the antimicrobial activity [110]. In 2006, Kumar et al. synthesized aminopyrimidines bearing benzofuran ring and evaluated them for their* in vitro* antibacterial activity against* P. aeruginosa *and* S. aureus* and antifungal activity against* A. niger* and* Curvularia*. All compounds (**71**) showed comparable antimicrobial activities with chloramphenicol and fluconazole which were used as standards for comparison of antimicrobial and antifungal activity, respectively [111]. Recently reported morphilino pyrimidines were synthesized by Kanagarajan et al. under microwave irradiation and screened for their* in vitro *antibacterial activity against* B. subtilis*,* Bacillus cereus*,* Micrococcus luteus*,and* Salmonella typhi* and antifungal activity against* A. niger*,* Candida 6*, and* Candida 51*. Structure activity relationship results for the synthesized compounds showed that compound (**72**) which has electron withdrawing chloro, fluoro, bromo, and nitro functional groups at the* para/meta *position of phenyl rings attached to C-4 and C-6 carbons of pyrimidine moiety delivered excellent antibacterial and antifungal activities [112] (Figure 11).

In 2007, Naik and Chikhalia synthesized a series of 2-[{2-(Morpholino)-3-pyridinyl-5-thio}-2-oxoethyl oxadiazolyl]-amino-4-(2,4-dichloro-5-fluoro phenyl)-6-(aryl)-pyrimidines and screened them for their antibacterial activity against* S. aureus*,* E. coli*,* S. typhi*, and* B. subtilis*. It was found that in comparison to standard drug compound (**73**) showed maximum zone of inhibition against* E.coli*,* S. aureus*,* S. typhi*, and* B. subtilis *[113]. A series of novel 2-amino-4-(1-napthyl)-6-aryl pyrimidines has been synthesized by Ingarsal et al. and evaluated for* in vitro* antibacterial and antifungal activities against representative bacteria—*S. aureus*,* E. coli*,* K. pneumonia*, and* P. aeruginosa* and fungi—*Trichophyton tonsurans and Microsporum gypseum*. It was found that compound (**74**) having 4-fluoro and 4-chloro groups had the best overall antibacterial and antifungal activities [114]. 4-(5-Bromo-1-benzofuran-2-yl)-6-(substituted phenyl)-pyrimidine-2-ol (**75**) and 4-(5-bromo-1-benzofuran-2-yl)-6-( substituted phenyl)-pyrimidine-2-thiol (**76**) were synthesized by Kumar et al. and subjected to* in vitro* antibacterial and antifungal activity. The compound possessed significant antibacterial activity against* B. subtilis*,* P. aeruginosa* and antifungal against* A. niger* and* C. albican* [115]. Recently, Ramesh and Sumana synthesized novel pyrimidines derivatives by combining chalcones of 2-acetyl thiophene with guanidine hydrochloride in the presence of alcohol and subjected the synthesised compounds to* in vitro* antibacterial and antifungal activity. It was found that, among the synthesized compounds, the compound (**77**) showed maximum antibacterial and antifungal activity at both 0.05 mL and 0.1 mL of concentration due to the presence of chloro and nitro groups as pharmacophores [116]. Patil et al. reported the synthesis of some new pyrimidines derivatives bearing paracetamol and imidazolyl moieties. The synthesized compounds (**78**) and (**79**) have been found to be active against* Rhizobium species*,* E. coli*,* Fusarium oxysporum*, and* Curvularia lunata *[117] (Figure 11).

**Figure 12:**
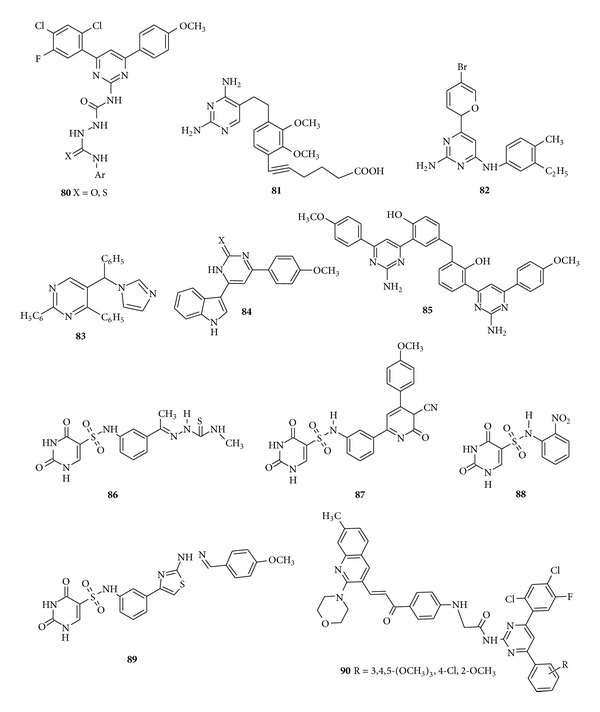
Trisubstituted pyrimidine derivatives of antibacterial and antifungal activity.

More recently, a novel series of 4-[4-(2,4-dichloro-5-fluorophenyl)-6-(4-methoxyphenyl) pyrimidine-2-yl]-1-(arylaminocarbnyl/thiocarbonyl)semicarbazides (**80**) were synthesized by Patel et al. and evaluated for their* in vitro* antibacterial activity against Gram-positive bacteria (*S. aureus *and* B. subtilis*) and Gram-negative bacteria (*E. coli *and* P. aeruginosa*). Results revealed that all the synthesized compounds exhibited excellent or moderate activity, also the substitution of electron withdrawing group on aromatic ring always optimizez the lead compound [118]. Data of antimicrobial activity of compound (**81**) synthesized by Forsch et al. revealed that the compound showed potent* in vitro* antibacterial activity in comparison to trimethoprim and was found to be 12,000 times more potent than trimethoprim and its potency actually exceeded that of trimethoprim by a factor of at least 10 [119]. A subsequent paper has checked out the* in vitro *antibacterial activity of 4-substituted 2-amino-6-(aniline) pyrimidines and screened them as selective inhibitors of DNA polymerase *Ι* *Ι* *Ι* in* S. aureus. *The compound (**82**) was found to be 5-fold more active than the prototype of 4-chloro compound having 97% inhibition because the presence of small hydrophobic substituents on the 3 and 4 positions of the aniline group combined with 4-halosubstituted phenoxy substituents was always found to increase the anti-Pol *Ι* *Ι* *Ι* activities of these Gram-positive selective inhibitors [120].* N,N*-Dimethyl-4-phenyl-5-[phenyl (*1H*-1,2,4-triazol-1-yl) methyl] pyrimidine-2-amine was synthesized by Menozzi et al. and screened for antibacterial activity. The compound (**83**) showed activity against* S. aureus* with MIC value 66 *μ*M and this was the only compound showing antifungal activity against* C. albicans* and* C. neoformans* due to the presence of pyrimidine nucleus bearing a double aromatic substitution at positions 2 and 4 [121]. Nassar synthesized the oxo- and thioxopyrimidines derivatives and screened them for their* in vitro* bactericidal activity against* S. aureus*,* E. coli*, and* P. aeruginosa. *The screened compound (**84**) showed high or moderate bactericidal activity compared to that of ciprofloxacin [122]. Novel 4-[3-(2-Amino-4-(4-chlorophenyl)pyrimidine-6-yl)-4-hydroxybenzyl]-2-(amino-4-(4-methoxyphenyl)pyrimidine-6-yl) phenol derivatives have been reported by Nagaraj and Reddy in 2008 and the compound (**85**) was found to be highly active against pathogenic bacteria* E. coli*,* S. aureus*, and* B. subtilis* and fungi* C. albicans* due to the presence of methoxy group at position 4 [123] (Figure 12).

A novel series of 2-thiouracil-5-sulfonamide derivatives were synthesized and investigated for* in vitro* antibacterial, antifungal, antiviral, and antitumour activities. Outcome of Structure Activity Relationship (SAR) study depicted the thiouracil nucleus as essential for the antimicrobial activity and substitution at position 5 of 2-thiouracil gave active compounds as antibacterial, antiviral, and antineoplastic; hence, the substituents group like thiourea in compound (**86**), pyridine moiety in compound (**87**), benzene nucleus with NO_2_ group in compound (**88**), and thiazole ring in compound (**89**) were found to show potent* in vitro* antimicrobial activity [124]. Chikhalia et al. in 2008 synthesized some newly substituted quinolinyl chalcones containing pyrimidines moiety and evaluated the* in vitro *antibacterial activity. The compound (**90**) and its various derivatives with an electron withdrawing group on the aromatic ring like 4-chloro or 2-methoxy and presence of more than one electron withdrawing group like 3,4,5-methoxy were found to show excellent activity against* S. aureus* and* B. subtillis *when compared with standard Ampicillin trihydrate [125] (Figure 12).

**Figure 13:**
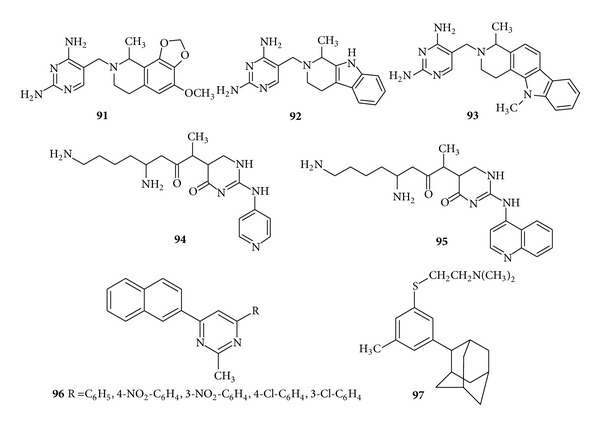
Trisubstituted pyrimidine derivatives of antibacterial and antifungal activity.

In another study, novel 2,4-diaminopyrimidines bearing N,N-disubstituted aminomethyl residues at the position 5 were designed as dihydrofolate reductase (DHFR) inhibitors. Results revealed that a number of compounds belonging mostly to the bi-(**91**), tri-(**92**) and tetra-(**93**) cyclic amine series showed a potent inhibitory activity mainly against TMP-sensitive and TMP-resistant DHFR from* S. pneumoniae* [126]. Some new derivatives of natural dipeptide antibiotics TAN 1057 A, B containing heterocyclic ring and  *β*-amino acid as a side chain were synthesized and screened for the* in vitro *antimicrobial activity by Brands et al. Results revealed that the amino pyridyl derivative, that is, compound (**94**), and amino quinolyl derivative, that is, (**95**), showed considerable antistreptococcal activity [127] (Figure 13).

With the view of potent antibacterial activity a new series of 4-(1-napthyl)-6-arylpyrimidine-2-(*1H*)-ones, (**96**), was synthesized by Vijayaramalingam et al. and evaluated for* in vitro* antibacterial activity. It was demonstrated from the data of* in vitro* antibacterial activity that there is an increase in activity profile when groups such as chloro, nitro, and fluoro are present in the phenyl ring [128]. Novel adamantylated pyrimidines were synthesized by Orzeszko et al. and compound (**97**) was found to possess significant* in vitro* antibacterial and antifungal activities against all the selected strains [129] (Figure 13).

**Figure 14:**
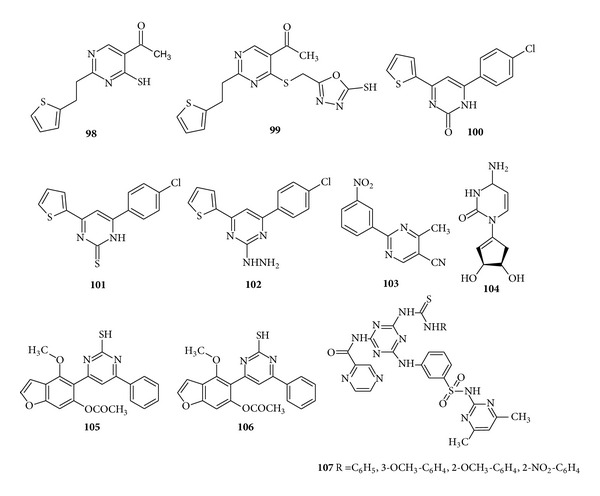
Trisubstituted pyrimidine derivatives of antibacterial and antifungal activity.

In the year of 2008, Moustafa et al. synthesized some new derivatives pyrimidines and selected members of the synthesized compounds were screened for* in vitro *antimicrobial activity. The compound (**98**) showed higher antibacterial activity than the standard drug (penicillin) while compound (**99**) showed higher activity against* C. albicans *than the standard drug (Nystatin) [130]. In another paper Ramiz et al. reported the synthesis of 2, 6 disubstituted pyrimidine derivatives and evaluated their* in vitro *antibacterial activity. It was demonstrated from the SAR studies that the oxo analogue of pyrimidine, that is, compound (**100**), showed less activity than the corresponding substituted thiopyrimidine derivatives; that is, compound (**101**) and 2-hydrazinyl dihydropyrimidine derivative (**102**) exhibited the highest activity against* B. subtilis*,* P. aeruginosa*, and* Streptomyces *species, with MIC values of 75 *μ*g/mL [131] (Figure 14).

Utilising 2-ethoxymethylene-3-oxobutanenitrile Černuchová et al. synthesized the pyrimidines derivatives. All the synthesized compounds displayed biological activity against bacteria, filamentous fungi, and tumour HeLa cells and the widest antimicrobial activity has been manifested by the compound (**103**) containing biologically active subunit: a nitro group in position 3 on the phenyl ring against* B. subtilis*,* S. aureus*, and* A. niger *[132]. Further, a series of truncated carbocyclic pyrimidine nucleosides were synthesized and evaluated for the* in vitro* antibacterial activity. The compound (**104**) (1′S, 2′R, 3′S)-1-[(2′,3′-dihydroxy)-4′-cyclopenten-1′-yl] cytosine was found to be the most active against the selected bacterial strains by inhibiting the S-adenosyl-homocysteine (SAH) hydrolase enzyme [133]. Another prominent work was reported by Micky et al. who synthesized the 1-arylethylene benzofuranyl ketone derivatives having pyrimidine moiety and tested their* in vitro *antibacterial activity. The compounds (**105**) and (**106**) due to the presence of pyrimidine-2-thione moiety were found to show moderate activity against two strains of bacteria* B. cerens *(Gram positive) and* E. coli* (Gram negative) by agar diffusion technique [134]. Patel and Gorgamwala reported the synthesis of 2-(pyrazine-2-carboxamido)-4-(2–*o*-aminobenzene sulfonamide-4,6–dimehylpyrimidine)-6-arylthiouredio)-s-triazine derivatives and screened them for* in vitro *antibacterial activity against* S. aureus *and* E. coli*. Amongst the synthesized derivatives compound (**107**) with the presence of phenyl ring and –CH_3_ at positions 3 and 4 on the phenyl ring, respectively, showed good activity and the presence of –OCH_3_ at position 2 displayed good activity while its presence at the fourth position and NO_2_ group on the second position of phenyl ring displayed slight to moderate activity [135] (Figure 14).

**Figure 15:**
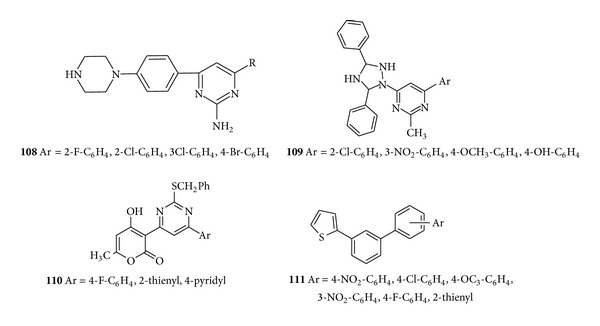
Trisubstituted pyrimidine derivatives of antibacterial and antifungal activity.

For the treatment of commonly occurring microbial disease Rahman et al. synthesized some new 2-amino-4,6-diarylpyrimidine derivatives (**108**) by the condensation of chalcone derivatives with guanidine hydrochloride and screened them for* in vitro antimicrobial activity* against* Bacillus subtilis*,* Bacillus pumilus*,* Proteus vulgaris*, and* E.coli*. Among the synthesized pyrimidine derivatives particularly halogen substituted derivatives showed more activity when compared with the standard drug ciprofloxacin [136]. Later, Khanage et al. described an efficient method for the synthesis 6-(substituted aryl)-4-(3,5-diphenyl)-1*H*-1,2,4-triazol-1-yl)-1,6-dihydropyrimidine-2-thiol (**109**) and screened the synthesized compounds for their* in vitro* antimicrobial activity by agar well method at 50 *μ*g/mL and 100 *μ*g/mL concentrations. Exclusively studied SARs revealed that the presence of methoxy, chloro, nitro, and hydroxyl substitution on phenyl ring of the pyrimidine nucleus produced remarkable* in vitro* antimicrobial activity against* S. aureus*,* E. coli*,* C. albicans*, and* A. niger* [137] (Figure 15).

Pyrimidine derivatives bearing a pyronyl side in the position 4 have been synthesized by using commercially available dehydroacetic acid (DHA) aromatic aldehydes and S-benzylthiouronium chloride (SBT) and tested for* in vitro *antimicrobial activity. Among the synthesized derivatives, compound (**110**) showed mild activity against Gram +ve* Bacillus subtilis* bacteria and minor activity against Gram −ve* P. aeruginosa *[138]. A novel series of 2-mercaptopyrimidines (**111**) were synthesized by combining (E)-thienyl chalcones with thiourea in alcohol medium and evaluated for their* in vitro* antibacterial and antifungal activities against Gram-positive organisms (*E. faecalis and S. aureus*),Gram-negative organisms (*K. Pneumoniae and E. coli*), and fungi (*C. albicans and A. niger*), also compared with that of the standard drugs ciprofloxacin and fluconazole. The antitubercular activity was carried out by using Middle brook 7H9 agar Blue Assay (MABA) method. The antibacterial results revealed that compounds showed significant activity against Gram-positive organisms and moderate activity against the Gram-negativeorganisms when compared with the standard drug and showed significant antitubercular activity with MIC ranging from 0.8 to 6.25 *μ*g [139] (Figure 15).

#### 5.1.3. Tetrasubstituted Pyrimidine Derivatives of Antibacterial and Antifungal Activities

**Figure 16:**
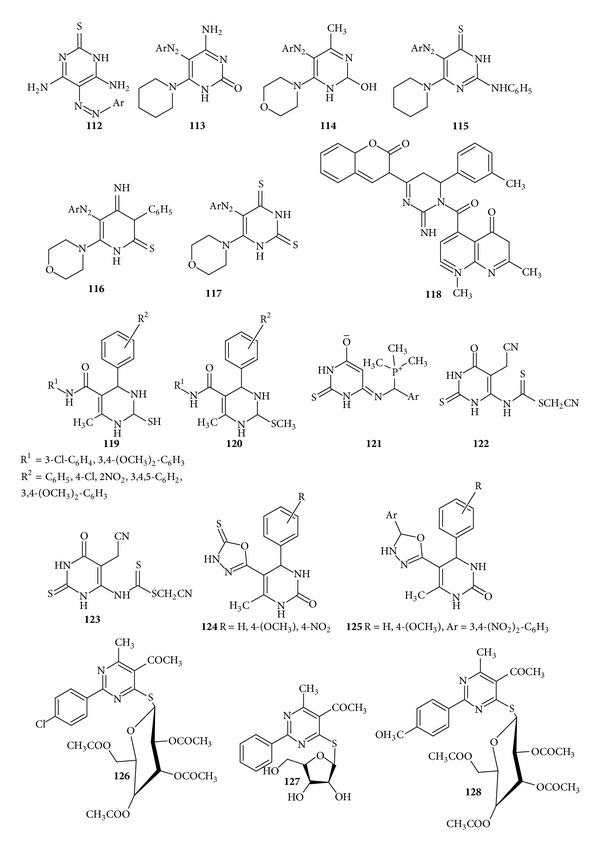
Tetrasubstituted pyrimidine derivatives of antibacterial and antifungal activity.

A variety of pyrimidines derivatives were synthesized by Aly and Nassar by utilizing* N*-(dicyclomethylazo) phenyl]-2-saccharin-2-ylacetamide and evaluated the derivatives for* in vitro* antibacterial activity and results revealed that compounds (**112**–**117**) showed promising activity towards bacteria [140]. Some novel series of pyrimidines derivatives were also reported by Waheed et al. and screened for their* in vitro *antibacterial activity. It is found that all the compounds were effective against Gram-negative test and compound (**118**) having bromo substituent on the* meta* position of aminopyrimidines showed appreciable activity against* E. coli *[141]. Parmar and Parikh reported the synthesis of some novel derivatives of pyrimidinethiones and the products have been screened for their* in vitro *growth inhibitory activities against several microbes like* Bacillus megaterium*,* B. subtilis*,* E. coli*,* Pseudomonas Fluorescens*, and* Aspergillus awamori*. It was found that the compounds (**119**) and (**120**) exhibited the significant activity [142]. Khir Eldin et al. utilized the 6-amino-2 thiouracil for the synthesis of several heterocyclic derivatives and examined the* in vitro* antibacterial activity of some products. It is found that the phosphorous containing derivative (**121**) showed the highest activity and the compounds (**122**) and (**123**) showed the moderate activity [143]. Purohi et al. reported the synthesis of the 5-[5′(mercapto)]-1,3,4-oxadiazol-2′-yl,6-methyl-4-aryl-3,4-dihydro pyrimidine2(1*H*) ones and 5-[5′-(aryl)]-1,3,4-oxadiazol-2′-yl,6-methyl-4-aryl-3,4 dihydro pyrimidine 2(1*H*)-ones and evaluated them for the the* in vitro *antimicrobial activities of the compounds. It was found that in comparison to the standard drug the compounds (**124**) and (**125**) exhibited the highest activity against* S. aureus *and* E.coli* and moderate activity against fungal organisms [144] (Figure 16).

Some new derivatives of S-*β*-D-glucosides 4-mercaptopyrimidine were synthesized by Moustafa et al. and evaluated for* in vitro *antimicrobial activity. Results revealed that the compound (**126**) showed the higher activity against* B. subtilis *(Gram-positive) and* E. coli* (Gram-negative) while the compound (**127**) showed good activity towards fungi whereas compound (**128**) showed moderate activity [145] (Figure 16).

**Figure 17:**
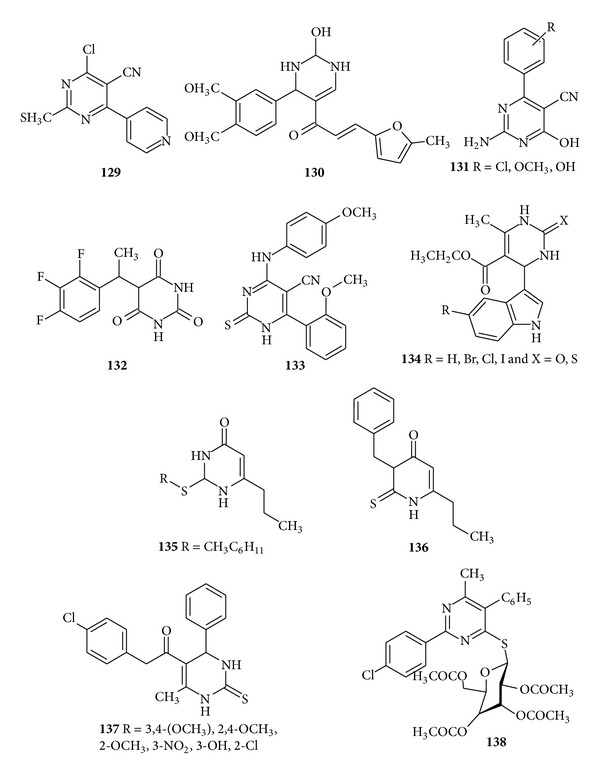
Tetrasubstituted pyrimidine derivatives of antibacterial and antifungal activity.

Upadhyay and Ram synthesized some pyrimidines and azolopyrimidines and evaluated the biodynamic properties of the synthesized compounds and found that the compound (**129**) was the most potent amongst all and inhibited the* in vitro* growth of the microbes up to 88% [146]. New derivatives containing 5-acetyl-4(3,4-dimethoxyphenyl)-6-methyl 2-oxo- 1,2,3,4-tetrahydropyrimidine moiety incorporated with different biologically active heterocycles such as pyridon, iminopyridines, thiazolidinones, and chalcones and 2-alkoxypyridines derivatives were synthesized and screened for their* in vitro* antibacterial and antifungal activity by El-Fattah et al. The compound (**130**) in 100 *μ*g/mL gave the highest antibacterial activity against all the tested strains with a mean zone of inhibition equal to 8.5 mm [147]. Moustafa et al. synthesized and evaluated the 2-amino-5-cyano-6-hydroxy-4-phenyl pyrimidines against Gram-positive and Gram-negative bacteria. The synthesized compound (**131**) was found to be selectively active against Gram-positive* S. aureus* bacteria; also substitution on the aromatic aldehydes reduces the activity [148]. Tomašić et al. synthesized 5-(3,4,5-trifluorobenzylidene) pyrimidine-2,4,6 (*1H,3H,5H*)-trione and tested it for* in vitro* antibacterial activity. The compound (**132**) had a great influence on antibacterial activity since 2,3,4-trifluorobenzylidenebarbituric acid possessed activity against* S. aureus* and mehticillin-resistant* S. aureus* with minimum inhibitory concentration (MIC) values of 32 *μ*g/mL and 128 *μ*g//mL, respectively [149]. The significant antimicrobial activity was found in 4-[(acetylphenyl) amino]-6-(2-methoxyphenyl)]-2-thioxo-1,2-dihydropyrimidine-5-carbonitrile derivatives when tested for their* in vitro* antimicrobial activity by Fathalla et al. Results revealed that the compound (**133**) possessed the highest antibacterial activity against* E. coli*,* Salmonella typhimurium*,* Listeria monoctyogenes*,* S. aureus*,* P. aeruginosus*, and* Bacillus cereus *[150] (Figure 17).

The novel series of indole dihydropyrimidine derivatives (**134**) were synthesized by Heda et al. and screened for their* in vitro* antibacterial activity against* E. coli and P. aeruginosa* and* in vitro* antifungal activity against* A. niger* and* Fusiform oxyporum*. The MIC was determined which showed promising results when compared with the standard drug [151]. The novel series of substituted 6-n-propyl thiouracils was synthesized by Prachayasittikul et al. and screened for* in vitro* antibacterial, antimalarial activities and as cytotoxic agents. The results showed that the compounds (**135**) and (**136**) exhibited complete growth inhibition against* S. pyogenes, B. catarrhalis*, and* M. lutens* with a minimum inhibitory concentration ranges of 32–128 *μ*g/mL [152]. Akbari et al. synthesized new N-(4-chlorophenyl)-6-methyl-4-aryl-2-thioxo-1,2,3,4-tetrahydropyrimidine-5-carboxamide (**137**) and evaluated its* in vitro* antibacterial and antifungal activities against* S. aureus*,* S. epidermidis*,* E. coli*,* P. aeruginosa*, and* C. albicans*, respectively. All the synthesized compounds exhibited moderate activity as compared to the standard [153]. In another work, Moustafa et al. synthesized some S-and N-*β-D-*glucosides derivatives of pyrimidine-4-thiol and evaluated the* in vitro* antibacterial activity. The compound (**138**) showed higher activity against* B. subtilis* as Gram +ve and* E. coli* as Gram −ve bacteria [154] (Figure 17).

**Figure 18:**
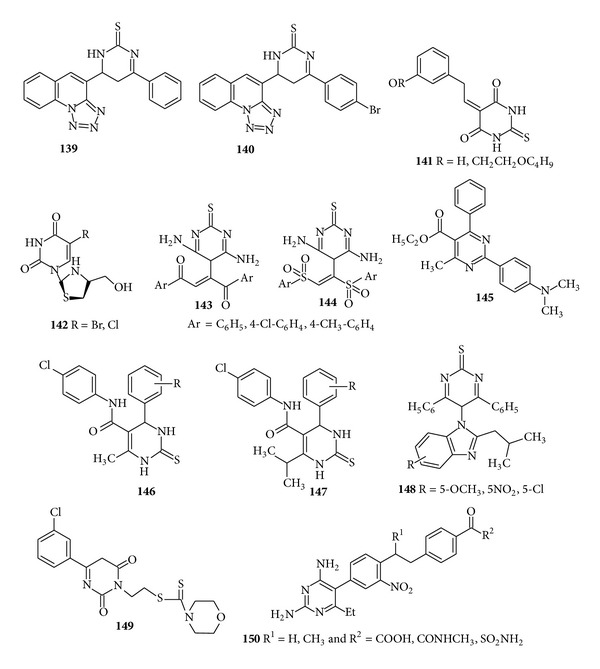
Tetrasubstituted pyrimidine derivatives of antibacterial and antifungal activity.

Three novel series of tetrazolo [1,5-a] quinoline derivatives containing pyrimidine moiety were synthesized by Bekhit et al. and screened for their* in vitro* antibacterial activity. The activity of compounds (**139**) was 50% of that of ampicillin against* E. coli* and the compound (**140**) showed activity against* S. aureus* comparable or half-fold of that of ampicillin [155]. As a result of pioneering work of Qin Yan, a novel series of 5-benzylidene thiobarbiturates (**141**) derivatives have been synthesized and screened for their antibacterial activity. These compounds exhibited selectively antibacterial activity against* S. aureus* with the MIC value of 3.1 *μ*g/m [156]. A new class of 1,3-thiazolidine pyrimidine nucleoside analogues were synthesized and evaluated by Sriharsha et al. for the* in vitro* antibacterial activity. The compound (**142**) due to the presence of free NH group in the pyrimidine moiety along with chloro or bromo substitution was always found to show increased and significant activity when compared with bacitracin and ciprofloxacin [157]. A new class of thiopyrimidine derivatives was synthesized by Padmaja et al. and evaluated for its* in vitro* antibacterial activity against Gram +ve and Gram −ve bacteria. The compounds (**143**) and (**144**) exhibited moderate to high activity towards Gram +ve bacteria with an inhibitory zone of 17–22 mm and moderate activity towards Gram −ve bacteria with an inhibitory zone of 18–25 mm [158]. Ethyl 2-(4-(dimethylamino)-4-methyl-6-phenylpyrimidine-5-carboxylate was synthesized regioselectively and evaluated for its* in vitro* antibacterial activity by Gholap et al. The compound (**145**) with no substituents on the phenyl ring showed more potent activities having MIC 16 *μ*g/mL which is comparable to that of the standard erythromycin [159] (Figure 18).

A series of dihydropyrimidines (DHPM) derivatives were synthesized and screened for the* in vitro* antibacterial activity. The compounds (**146-147**) were found to have significant zone of inhibition against* E.coli*,* E. aerogenes*,* Bacillus spizizenii*,* P. aeruginosa*,* E. coli*, and some other strains [160]. A new series of 5-[2-(2-methylpropyl-enyl)-1*H* benzimidazolyl-4,6-diphenyl-pyrimidin-2-(5H)-thione (**148**) derivatives have been synthesized and subjected to evaluation of their* in vitro* antibacterial activity against* B. subtilis*,* S. aureus*,* E. coli*, and* Pseudomonas diminuta *by Sharma et al. It was deduced from the data that the compounds containing electron withdrawing groups, that is, nitro, chloro, and methoxy substitution, were found to show enhanced and remarkable antibacterial activity [161]. Septioğlu et al. synthesized some new* N,N-*disubstituted dithiocarbamic acid 2-(6-arylhexahydropyrimidine-2,4-dione-3-yl) ethyl esters derivatives and evaluated their* in vitro* antibacterial activity against* S. aureus*,* E. faecalis*,* E.coli*, and* P. aeruginosa* and antifungal activity against* C. albicans*,* Candida krusei*, and* Candida parapsilosis* and found that the compound (**149**) having MIC value 16 *μ*g showed the most favourable antibacterial activity [162]. A few years later in 1997, Robson et al. synthesized novel 2,4-diamino-5-(4′-benzylamino) and 2,4-diamino-5-[4′-N-methylbenzylamino)-3′-nitrophenyl]-6-ethylpyrimidines bearing 4-substituents on the benzylamino or N-methyl-benzylamino aryl ring and evaluated them for their antibacterial activity. Structure activity relationship study from the given data revealed that the compound (**150**) with the introduction of* para* substituent like COOH, CONH_2_, CONHMe, and SO_2_NH_2_ exhibited potent inhibitory activity against* Toxoplasma gondii* as compared to benzoprim or methylbenzoprim [163] (Figure 18).

**Figure 19:**
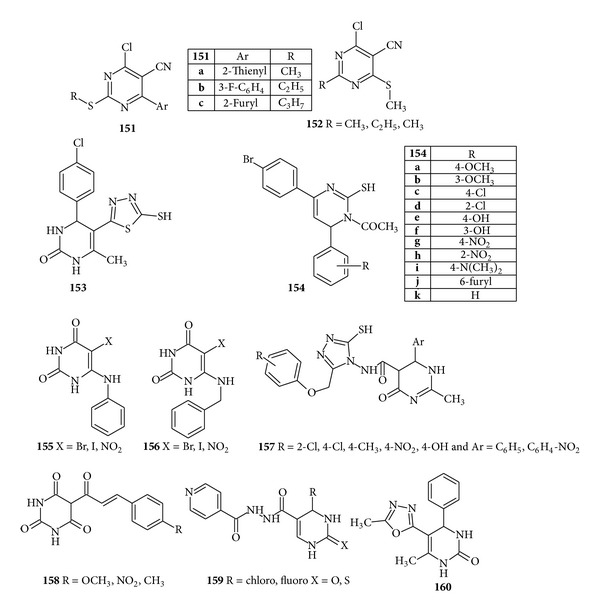
Tetrasubstituted pyrimidine derivatives of antibacterial and antifungal activity.

Although chloropyrimidines appeared to have effective antimicrobial activity, Agrawal et al. synthesized some new derivatives containing aryl, heteroaryl, and alkylthio substituents at position 6 and also alkylthio substituents at position 2 because structure activity data of chloropyrimidine revealed that substitution at positions 2 and 6 has great influence on antitubercular activity; hence, synthesized derivatives were screened for* in vitro* antibacterial, antitubercular, and antifungal activities. The compound (**151**) with its various derivatives was found to display good antibacterial and antitubercular activity with MIC value 0.78 *μ*g/mL and compound (**152**) demonstrated a good antifungal activity when compared with the standard drug clotrimoxazole [36] (Figure 19).

In the hope of potent antifungal activity some pyrimidinone derivatives were synthesized and screened for* in vitro *antifungal activity and compound (**153**) demonstrated the significant activity against* Phytophthora infestans *and* Colletotrichum falcatum* with the introduction of chloro group [164]. A new series of 4(4′-bromophenyl)-6-substituted aryl-1-acetylpyrimidine-2-thiol derivatives were synthesized and screened for the* in vitro *antimicrobial activity. The results showed that all the synthesized compounds (**154**) have shown significant, good to moderate antimicrobial activity due to the presence of thio group, which enhanced the respective activities with varied substituent groups, against selected pathogens [165]. Trantolo et al. synthesized a series of N^6^-substituted 6-aminopyrimidines and examined the effects of substituents governing the inhibition of DNA polymerase *Ι* *Ι* *Ι* from* B. subtilis. *The compounds (**155**) and (**156**) having bromo and iodo substituents on position 5 of 6-(benzylamino) uracils and 6-p-toluidinouracils were equipotent with the parent compound [166]. However, Shivkumar et al. also reported some pyrimidinone derivatives and evaluated their* in vitro *antifungal activity. Results indicated that the compound (**157**) showed significant activity against* C. albicans* [167] (Figure 19).

A series of eleven* 5-((E)-3-phenylacryloyl) pyrimidine-2,4,6-(1H,3H,5H)-triones (5-acetyl pyrimidine 2,4,6-(1H,3H,5H) trione based chalcones)* were synthesized and screened for antibacterial and antifungal activities by Dhorajiya et al. Results revealed that compound (**158**) was comparable with the standard drug ciprofloxacin in case of* P. aeruginosa* [168]. For developing some new antimicrobial drugs, Elumalai et al. in the year of 2013 developed some new isoniazid cyclocondensed 1,2,3,4-tetrahydropyrimidine derivatives and evaluated them for* in vitro* antimicrobial and antimycobacterial activities against* Bacillus subtilis*,* E. coli*,* Mycobacterium tuberculosis CIP*, and* H*_*37*_ *Rv* strain. Exclusively studied SARs revealed that 1,2,3,4-tetrahydropyrimidines contain substituted phenyl and hetero aromatic rings responsible for antimicrobial and antimycobacterial activities. Substituted atom or group of atoms must be of strong electron withdrawing nature for potent activity because it decreases electron density in the ring due to inductive effect. Substitution of fluoride and chloride substitution at fourth and third positions of phenyl ring showed potent activity; also introduction of heterocyclic ring at fourth position showed moderate activity. Hence it was concluded that compound (**159**) was arguably the most potent when compared with current therapeutic agents norfloxacin and rifampicin because fluoride and chloride substituted phenyl ring present at 4th position of 1,2,3,4-tetrahydropyrimidines enhancing the antimicrobial and antimycobacterial activities [169]. In the recent 2013, a series of pyrimidine bearing 1,3,4-oxadiazole derivatives have been synthesized and evaluated for antifungal activity against* Candida albicans, Penicillium *spp., and* Aspergillus niger*. It was found that compound (**160**) has shown promising antifungal activity at 10 *μ*g/mL concentration when compared with the standard drug amphotericin-B [170] (Figure 19).

### 5.2. Antimycobacterial Activity of Pyrimidines

From the study of vast literature it was found that pyrimidine derivatives possessing antimycobacterial activity consist of only trisubstituted and tetrasubstituted pyrimidines derivatives.

#### 5.2.1. Trisubstituted Pyrimidine Derivatives with Antimycobacterial Activity

**Figure 20:**
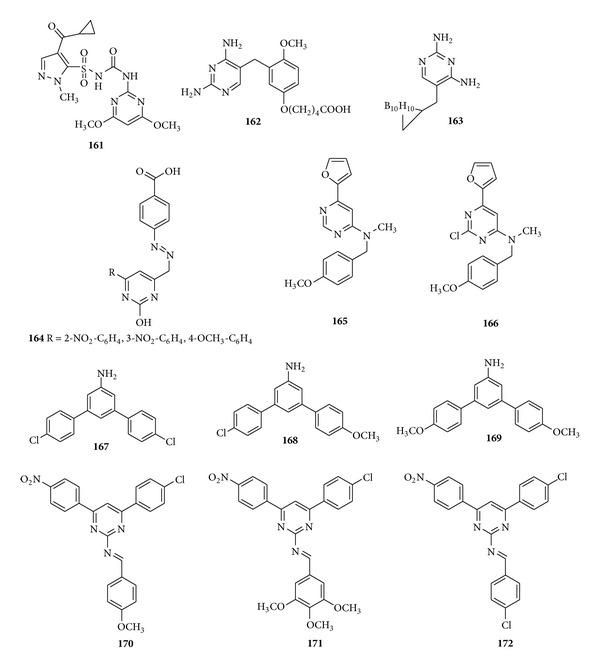
Trisubstituted pyrimidine derivatives of antimycobacterial activity.

Sohn et al. reported a paper in which around 100 anologues of sulfonylureas were screened for antimycobacterial activity against* M. tuberculosis*. The compound (**161**) was found to show comparable antimycobacterial activity with that of sulfometuron methyl inhibitor of acetohydroxyacid synthase (AHAS) and after demonstrated SAR information it was concluded that both 2-alkylcarbonyl and 2-hydroxyalkyl with 6-ethythio substituents at the aromatic backbone might be a good scaffold in the sulfonylureas possessing potent activity [171]. 2,4-Diamino-5-[2-methoxy-5-(*ω*-carboxyalkoxy) benzyl] pyrimidines derivatives were synthesized by Rosowsky et al. and tested for their ability to inhibit partially purified foam* P. carinii*,* T. gondii*, and* M. avium*. 5′-*O-*(*ω*-Carboxyalkyl) analogs were found to be the most potent and the compound (**162**) was found to be 245 times more potent than trimethoprim against Pc and 33-fold higher than that of trimethoprim against* Mycobacterium avium* [172]. Also, 2,4-diamino-6-methylpyrimidine containing 5-(7,8-nido-dicarbaundecarboran-7-yl) methyl or 5-(1,2-closo-dicabadodecarboran-1-yl) methyl substituents were synthesized and screened for their activity against* P. carnii*,* T. gondii*,* M. avium*,* M. tuberculosis*, and* Lactobacillus casei*. The compound (**163**) was found to show 10 times greater activity [173]. Trivedi et al. carried out the synthesis of some new pyrimidines derivatives via a novel chalcone series and evaluated them for their antimycobacterial activities against* M. tuberculosis* H_37_Rv. The compound (**164**) produced the highest efficacy and exhibited >90% inhibition at 6.25 *μ*g/mL due to the presence of 2-nitro, 3-nitro, and 4-methoxy substitution at phenyl ring added remarkable improvements in anti-tubercular activity [174] (Figure 20).

Pyrimidine analogs of antimycobacterial 6-aryl-9-benzylpurines were synthesized by Read et al. and screened for their* in vitro* antibacterial activity against* M. tuberculosis *H_37_Rv. Among the screened furylpyrimidines, the compounds having N-methylbenzylamino group showed better activities and the compounds (**165-166**) were identified as the most active compounds having minimum inhibitory concentration of 3.0 *μ*g/mL [175]. In another work, again some substituted pyrimidines were synthesized from chalcones by Umaa et al. and evaluated for their antitubercular potential and we found that three compounds (**167**–**169**) exhibited significant activity being equipotent with the standard drug [176]. A variety of novel 4-(4-substituted phenyl)-6-(4-nitrophenyl)-2-substituted imino) pyrimidines were reported by Siddiqui et al. and we investigated their antiviral, antitubercular, and antibacterial activities. Results indicated that the pyrimidines with chloro and p-methoxyphenyl (**170**), trimethoxy (**171**) showed more potent activity than the reference drug and pyrimidines with chloro and chlorophenyl (**172**) and* p*-N-dimethylaminophenyl substitutions showed equipotent activity in comparison with that of reference drugs [177] (Figure 20).

#### 5.2.2. Tetrasubstituted Pyrimidine Derivatives Having Antimycobacterial Activity

**Figure 21:**
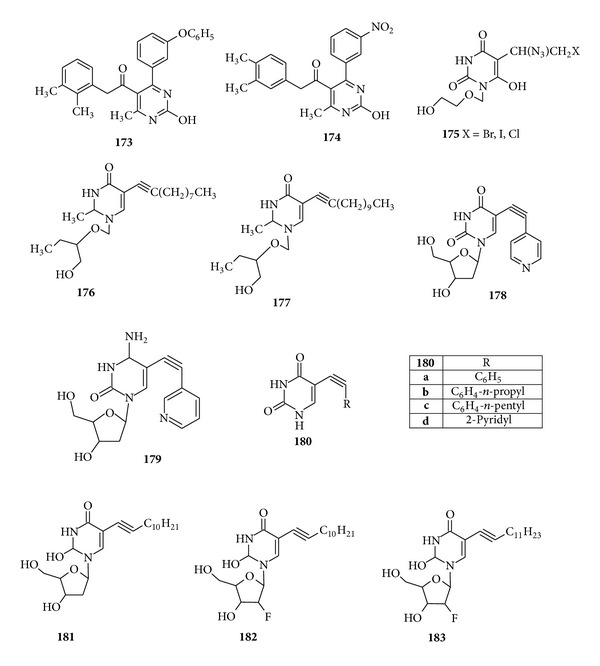
Tetrasubstituted pyrimidine derivatives of antimycobacterial activity.

N-Phenyl-6-methyl-2-oxo-4-phenyl-1,2,3,4-tetrahydropyrimidines-5-carboxamides derivatives were synthesized by Virsodia et al. and evaluated for their antitubercular activity against* Mycobacterium tuberculosis* and we found that compounds (**173**) and (**174**) with 2,3-dimethylphenyl and 3,4-dimethyl carbomyl side chains, respectively, showed 65% and 63% inhibition; hence, presence of methyl groups on phenyl ring of C5 side chain with* meta*-substituted 4-phenyl was found to show good activity [178]. Srivastav et al. evaluated the antimycobacterial activities of 1-(2-hydroxyethoxy)methyl-5-(1-azido-2-haloethyl or 1-azidovinyl) analogs and 1-[(2-hydroxy-1-(hydroxymethyl) ethoxy)methyl-5-decynyluracil and 1-[(2-hydroxy-1-(hydroxymethyl) ethoxy)methyl]-5-dodecynluracil against* M. tuberculosis*,* Mycobacterium bovis*, and* Mycobacterium avium*. It was found that 5-substituted acyclic pyrimidine compounds, that is, (**175**–**177**), were found to exhibit significant* in vitro* antimycobacterial activity [179] (Figure 21).

Srivastav et al. investigated the* in vitro *antimycobacterial activities of 5-alkyl, 5-alkynyl, furanopyrimidines, and 2′-deoxynucleosides derivatives. It was found that compounds with 5-arylalkynyl substituents, that is, (**178**–**180**), displayed potent* in vitro *antitubercular activity against* M. bovis *and* M. tuberculosis *[180]. Johar et al. synthesized a series of 1-*β-*D-2′-arabinofuranosyl and 1-(2′-dedoxy-2′-fluoro-*β*-D-ribofuransoyl) pyrimidine nucleosides possessing a diverse sets of alkenyl, alkyl, and halo substituents at the C-5 position of the uracil and investigated their effect on activity against* M. tuberculosis*,* M. bovis*,* and M. avium*. From a comprehensive overview and thorough study of SAR it is suggested that the compounds of 5-alkynyl series, that is, (**181**–**183**), showed the highest antimycobacterial potency which was similar or close to that of the rifampicin [181] (Figure 21).

### 5.3. Antimalarial Activity of Pyrimidines

Pyrimidine derivatives which were known to have antimalarial activity mainly consist of trisubstituted derivatives.

#### 5.3.1. Trisubstituted Pyrimidine Derivatives of Antimalarial Activity

**Figure 22:**
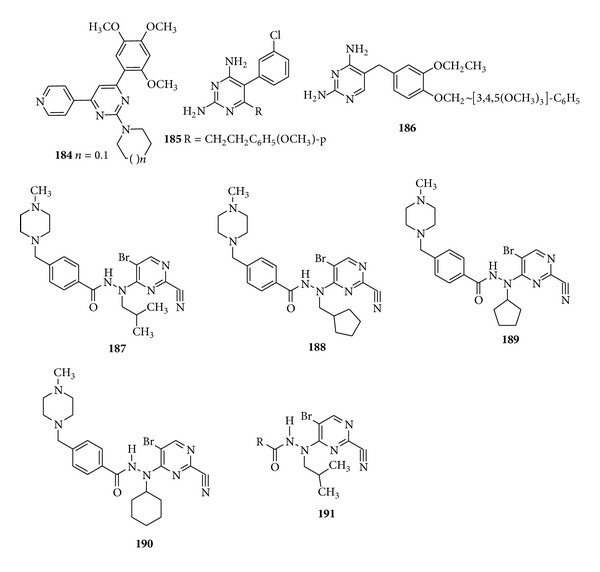
Trisubstituted pyrimidine derivatives possessing antimalarial activity.

A series of 2,4,6 trisubstituted-pyrimidines (**184**) were synthesized and evaluated for their* in vitro* antimalarial activity. Out of total screened compounds, 14 compounds have shown* in vitro* antimalarial activity against* Plasmodium falciparum* with MIC in the range of 0.25–2 *μ*g/mL and SAR study revealed that the substitution on phenyl ring shows an increased activity as substitution of methyl group at the* para* position of the compound represents a remarkable increase in activity [182]. The novel pyrimethamine and trimethoprim analogs were developed and tested for the* in vitro* antimalarial activities of synthesized compounds against* P*.* falciparum. *Pyrimethamine analogues (**185**) bearing* m*-Cl and unsubstituted 5-phenyl group together with long 6-alkyl substituents and trimethoprim analogues (**186**) were 10–25 times more effective than their parent compounds [183] (Figure 22).

Recently, the optimization studies of the 2-pyrimidine carbonitrile as lead series were done by Coterón et al. who studied the different heteroarylnitrile derivatives as potential falcipain inhibitors and therefore, potential antiparasitic lead compounds, with the 5-substituted-2-cyanopyrimidine chemical class emerging as the most potent and promising lead series. Results revealed that the introduction of a protonable amine in the structure of compound markedly improved the antiparasitic activity. The compounds (**187**–**190**) and derivatives of (**191**) presented excellent* in vitro *enzymatic activity and antiparasitic activity against* P. falciparum *[184] (Figure 22).

### 5.4. Antileishmanial Activity of Pyrimidines

#### 5.4.1. Monosubstituted Pyrimidine Derivatives of Antileishmanial Activity

**Figure 23:**
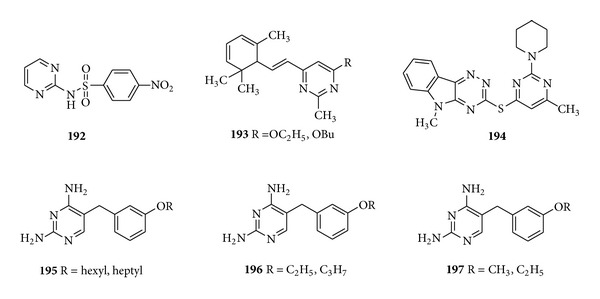
Monosubstituted (**192**) and trisubstituted (**193**–**197**) pyrimidine derivatives having antileishmanial activity.

A series of compounds containing the nitrobenzene and sulfonamido moieties monosubstituted pyrimidine derivatives were synthesized and their leishmanicidal effect was assessed* in vitro *against* L. infantum *promastigotes. Among the evaluated compounds, the* p*-nitrobenzenesulfonamides (**192**) showed significant activity due to the presence of electron withdrawing nitro group [185] (Figure 23).

#### 5.4.2. Trisubstituted Pyrimidine Derivatives Possessing Antileishmanial Activity

Some novel N- and O-substituted terpenyl trisubstituted pyrimidines derivatives were developed and screened for their* in vitro* antileishmanial activity profile in promastigote model by Chandra et al. The compound (**193**) with* p*-anisidino substitution showed 98.8% inhibition at a 1 *μ*g/mL concentration, whereas with OEt and OBu substitution the same compound showed 99% inhibition at 5 *μ*g/mL concentration [186]. A series of [1,2, 4] triazino[5,6-b]indol-3-ylthio-1,3,5-triazines and pyrimidines derivatives were synthesized and screened for their* in vitro* antileishmanial activity against* Leishmania donovani*. The pyrimidine derivatives (**194**) having piperidine at position 2 were found to show comparable activity against leishmaniasis with an IC_50_ of 4.01 and MIC of 20.54  *μ*M and to be the least toxic compounds which made them 20- and 10-fold more selective than that of the standard drugs pentamidine and stibogluconate [187]. A few years later in 1999, Chawdhury et al. synthesized compounds based on 5-benzyl-2,4-diaminopyrimidines and evaluated the compounds as inhibitors of leishmanial and trypanosomal dihydrofolate reductase. Results revealed that the compounds (**195**–**197**) were found to show good activity against* Trypanosoma brucei*,* Trypanosoma cruzi* and marginal activity against* Leishmania infantum* [188] (Figure 23).

### 5.5. *In Vitro *Antiamoebic Activity and Antiparsitic Activity of Pyrimidine

**Figure 24:**
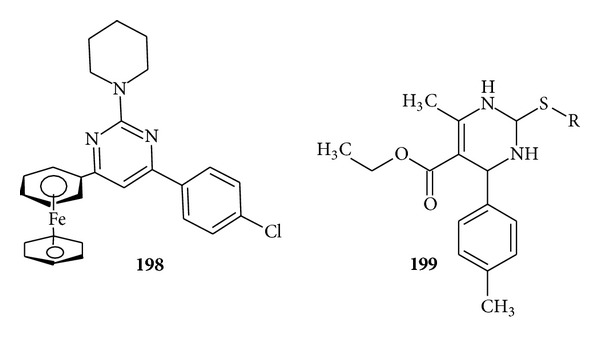
Trisubstituted pyrimidine derivatives with antiamoebic (**198**) and antiparasitic activity (**199**).

Praveen et al. synthesized a new series of 6-ferrocenyl aryl 2-substituted pyrimidines and evaluated them for their* in vitro* antiamoebic activity against HMI:1MSS strain of* Entamoeba histolytica*. Out of 16 compounds, 10 compounds have methoxy substitution, thio group and chloro group exhibited higher antiamoebic activity than the reference drug metronidazole, and compound (**198**) was found to be most active and least toxic [189] (Figure 24).

Some new tetrasubstituted pyrimidine derivatives were developed as a new class of antifilarial agents as a series of 2-sulfonyl-6methyl-1,4-dihydropyrimidines were synthesized by Singh et al. and evaluated for their antifilarial activity against adult parasites of human lymphatic filarial parasites* Brugia malayi in vitro *at various concentrations. The compound (**199**) showed promising antifilarial activity [190] (Figure 24).

### 5.6. *In Vitro* Antiviral Activity of Pyrimidines

To study* in vitro* antiviral activity of various pyrimidine derivatives, we have classified the pyrimidine containing derivatives on the basis of substitution and this substitution is as follows.

#### 5.6.1. Trisubstituted Pyrimidine Derivatives

**Figure 25:**
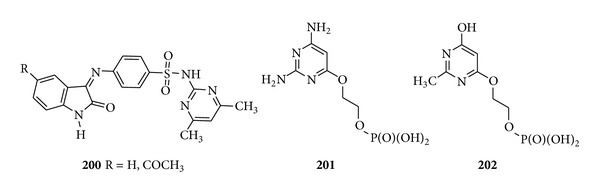
Trisubstituted pyrimidine derivatives of antiviral activity.

With the view of potency of sulfonamides Selvam et al. synthesized the 4-[(1,2-dihydro-2-oxo-3H-indole-3-ylidene)amino]-*N*-(4,6-dimethyl-2-pyrimidinyl)-benzene sulfonamide derivatives and carried out anti-HIV assay on them and found that the compound (**200**) was more active against the replication of HIV-1 and HIV-2 in membrane type 4 [MT-4] [191]. The discovery in 2002 by Holý et al. showed the antiviral activity of 6-[2-(Phosphonomethoxy) alkoxy] pyrimidines derivatives. Several analogues among the 6-[2-(phosphonomethoxy)ethoxy] (PMEO] pyrimidines derivatives showed pronounced antiviral activity in cell culture and the derivatives that carry an amino group at C-2 of the pyrimidine ring, that is, (**201**) and (**202**), emerged as the most potent compounds [192] (Figure 25).

#### 5.6.2. Tetrasubstituted Pyrimidines Derivatives

**Figure 26:**
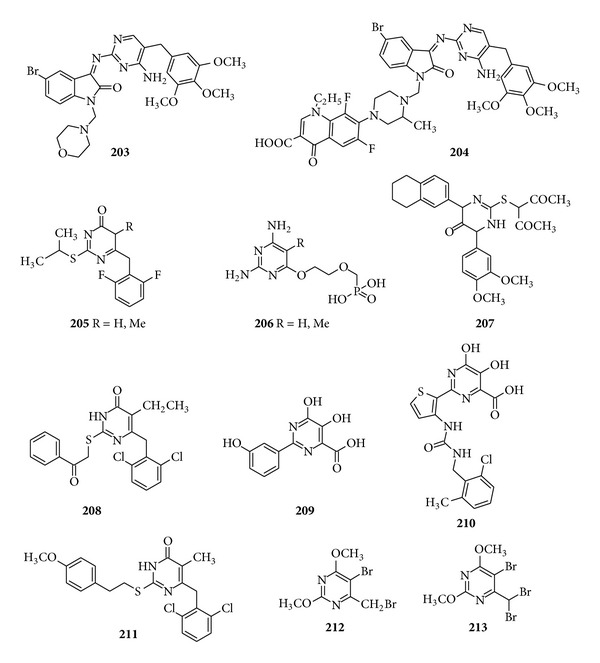
Trisubstituted pyrimidine derivatives of antiviral activity.

Sriram et al. designed the aminopyrimidinimino istain derivatives as a novel nonnucleosidase reverse transcriptase inhibitor with broad-spectrum chemotherapeutic properties for the effective treatment of AIDS (Figure S2). The compounds (**203**) and (**204**) with their different derivatives like ciprofloxacin moiety and lomefloxacin moiety at N-1 position emerged as the most potent broad-spectrum chemotherapeutic agent active against HIV, HCV,* M. tuberculosis*, and various pathogenic bacteria [193]. In the early 1999, the novel potent and selective dihydro-alkoxy-benzyl-oxypyrimidine derivatives, namely, 5-alkyl-2-(thio)-2,6-(-diphenylmethyl)-3,4-dihydropyrimidine-4(*3H*)-ones derivatives, were synthesized and tested as anti-HIV agent in both cell based and enzyme based assays. Result revealed that the compounds having 6-(2,6-difluorophenylmethyl) substituents (**205**) were found to be most potent significantly higher than that of nevirapine with EC_50_'s ranging between 40 and 90 *μ*M [194]. 2,4-Diamino-5-cyano-6-{[(diisopropoxyphosphoryl) methoxy] ethoxy} pyrimidines derivatives (**206**) were synthesized by Hocková et al. and evaluated for their* in vitro* antiviral activity and they found that 5-cyano and 5-formyl derivatives showed remarkable activity against herpes simplex virus type 1 or type 2,* cytomegalovirus*, and vaccinia virus with inhibitory concentration of 0.0027–0.011 *μ*mol/mL [195] (Figure 26).

A subsequent paper has been reviewed on antiviral activity in which Mohamed et al. prepared the 2-(2,4-dioxopentan-3-ylthio)-1,6-dihydro-4-(1,2,34-tetrahydronapthalen-6-yl)-6-(3,4-dimethoxyphenyl)pyrimidin-5(4*H*)-one derivatives and screened them for their* in vitro* antiviral activity and compound (**207**) was found to have good antiviral activity with 90% inhibition; hence, it was considered to be highly promising compound by confirming and comparing its activity with the antiviral activity of acyclovir [196]. A series of dihydro-alkylthio-benzyl-oxopyrimidines were synthesized by Nawrozkij et al. who evaluated the compounds for their* in vitro* antiviral activity. The compound (208) with 2,6-dichloro-, 2-chloro-6-fluoro-, and 2,6-difluoro-benzyl substitution at C-6 and ethyl, iso-propyl at C-5 was found to be the most potent derivatives due to its capability to inhibit the HIV-1 strain by 50% [197]. A novel and new class of the dihydroxypyrimidinecarboxylic acid derivatives and corresponding N-methylpyrimidinone were prepared by Summa et al. and tested in the HCV replicon assay. The compound (**209**) having the desired phenolic function in the* meta* position was found to be the most active having IC_50_ of 5.8 *μ*M [198]. Another paper reported the 2-(2-thienyl)-5,6-dihydroxy-4-carboxypyrimidines derivatives which were screened as inhibitors of the hepatitis C virus NS5B and the compound (**210**) showed the best cell based activity which was 60-fold much better than enzyme based assay and showed no cytotoxicity up to 100 *μ*M [199]. Manetti et al. carried out the parallel solution phase and microwave-assisted synthesis of dihydroxy-alkoxy-benzyl-oxypyrimidines (DABOs) and screened them for their antiviral activity toward both the highly purified recombinant human immunodeficiency virus type 1 (HIV-1) reverse transcriptase (RT) enzyme (wild-type and mutants) and wild-type (wt) and mutant HIV-1 strains. Results from anti-HIV reverse transcriptase assay and anti-HIV activity in lymphoid cells revealed that compound (**211**) had an anti-HIV profile comparable to that of nevirapine and efavirenz [200]. Novel C-6 fluorinated acyclic side chain pyrimidine derivatives were synthesized by Prekupec et al. and evaluated for their* in vitro* antiviral activity against* cytomegalovirus* (CMV^a^ AD-169 and Davis strain) in human embryonic lung (HEL) cells; Varicella-zoster virus, parainfluenza virus-3, retrovirus-1, Sindbis, and Punta Toro virus in vero cell. The 5-bromopyrimidine derivatives (**212**) and (**213**) showed the highest activity [201] (Figure 26).

## 6. Conclusion

As a result of vigorous research and vast literature survey, the dogma laid down by chemistry of pyrimidines has engendered long considerable interest which makes them occupy a very distinct and unique place in our lives. From the above review it was concluded that the trisubstituted and tetrasubstituted pyrimidines containing electron withdrawing group like nitro on the phenyl ring were found to show more potent* in vitro* antimicrobial activity as compared to the chloro or methoxy group. This offers the medicinal chemist continued interest in pyrimidine moiety in drug development against microbes. It will also ensure the development of reliable methods for the construction of important area of research in heterocyclic chemistry.

## Supplementary Material

Bleomycin a glycopeptide antibiotic produced by the bacterium Streptomyces verticillus. It is used as an antineoplastic and inhibits the DNA metabolism of the solid tumors.

## Abbreviations

MRSA: Methicillin-resistant* Staphylococcus aureus*
DHFR: Dihydrofolate reductase
AIDS: Acquired Immune Deficiency Syndrome
IDU: 5-Iodo-2′-deoxyuridine
CMV: Cytomegalovirus
SBT: S-Benzyl isothiouronium chloride
MIC: Minimum inhibitory concentration
DABOs: Dihydroxy alkoxy-benzyloxy pyrimidines
HIV-1: Human deficiency virus type 1
HEL: Human embryonic lung cells
RT: Reverse transcriptase.
