# Supplementary material for: Significance and Biological Importance of Pyrimidine in the Microbial World
Source: Int J Med Chem. 2014 Mar 23;2014:202784. doi: 10.1155/2014/202784 (PMC4207407; doi:10.1155/2014/202784)
Supplement: Supplementary file 1 — Bleomycin a glycopeptide antibiotic produced by the bacterium Streptomyces verticillus. It is used as an antineoplastic and inhibits the DNA metabolism of the solid tumors. [file 202784.f1.pdf]

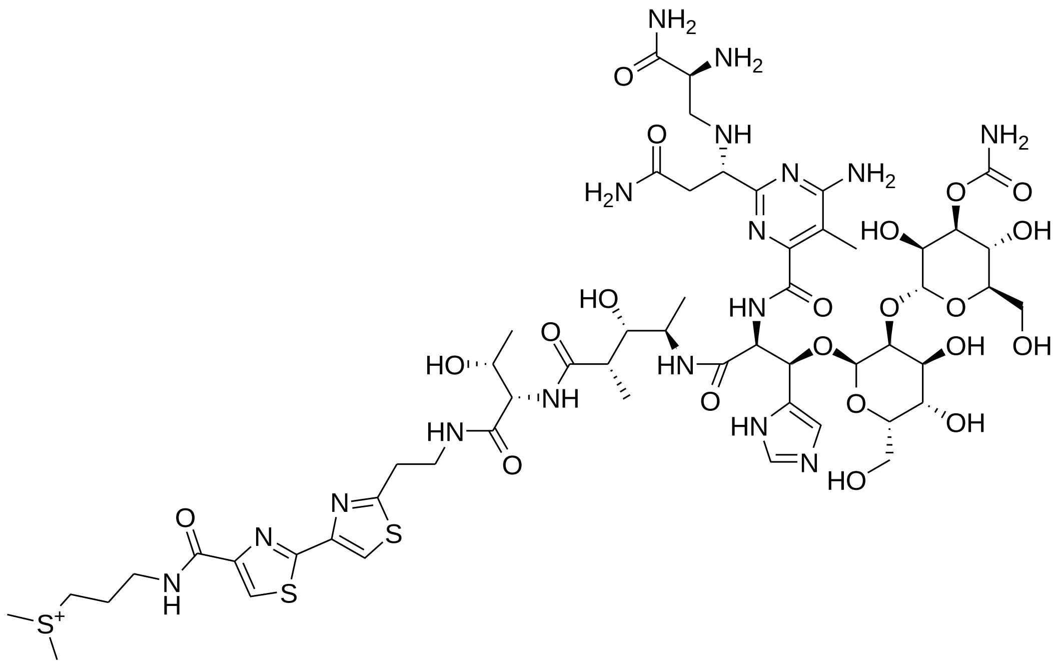

Figure S1 Bleomycin

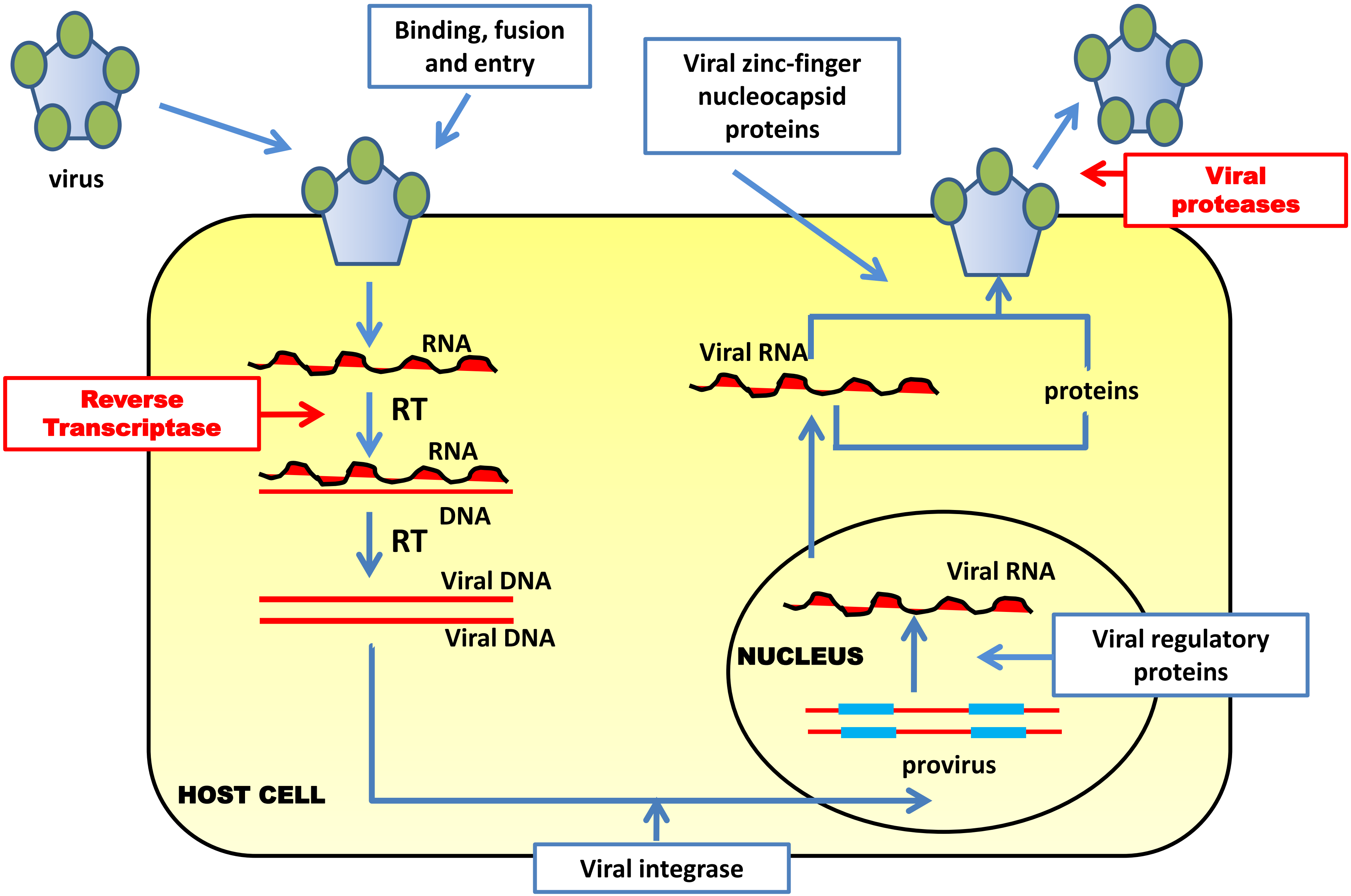

Figure S2 Antiviral actions of pyrimidine analogs ex. ganciclovir is phosphorylated first by viral kinase to the monophosphate. This intermediate are then phosphorylated by host cell kinase to the nucleotide analogs that inhibit viral replication.
